# Differential Susceptibility of *Coleomegilla maculata* and *Scymnus creperus* Larvae to Aggression by *Solenopsis invicta* Workers

**DOI:** 10.3390/insects14040318

**Published:** 2023-03-26

**Authors:** Eric W. Riddick, Zhixin Wu, Jian Chen

**Affiliations:** National Biological Control Laboratory, United States Department of Agriculture, Stoneville, MS 38776, USAjian.chen@usda.gov (J.C.)

**Keywords:** aphids, biological control, defense mechanism, ladybird beetles, predation, semiochemicals

## Abstract

**Simple Summary:**

The biological control of aphids by aphid-eating lady beetles (coccinellids) is often hampered by aphid-tending ants or aggressive, invasive ants foraging for food; aggressive species, such as the imported fire ant, may attack and kill lady beetle larvae. This study tested the hypothesis that wax-secreting coccinellid larvae are less susceptible than non-wax-secreting coccinellid larvae to fire ant aggression. Laboratory experiments were set up using bird cherry-oat aphid nymphs and adults (as prey for coccinellids) on barley leaves with either coccinellid species with or without fire ant workers. Results revealed that the presence of fire ants reduced aphid predation by non-wax-secreting but not wax-secreting larvae. The frequency of fire ant attacks was greater on non-wax-secreting larvae; the mortality was significantly greater for non-wax-secreting larvae. The wax covering over coccinellid larvae reduced fire ant aggression. Unexpectedly, coccinellid larvae with the wax cover removed did not suffer greater fire ant attacks or increased mortality. In conclusion, the wax cover and, perhaps, the volatile or non-volatile compounds in the wax and on the integument of wax-secreting coccinellid larvae reduce fire ant aggression. Further research could identify the wax compounds and determine their role as potential repellents or irritants against fire ants.

**Abstract:**

The biological control of aphids by aphidophagous coccinellids is often hampered by aphid-tending ants or aggressive, invasive ants foraging for food. Aggressive species such as the imported fire ant *Solenopsis invicta* Buren may attack and kill coccinellid larvae. This study tested the hypothesis that wax-secreting *Scymnus creperus* Mulsant larvae are less susceptible than non-wax-secreting *Coleomegilla maculata* (DeGeer) larvae to *S*. *invicta* aggression. Laboratory experiments were set up using bird cherry-oat aphid *Rhopalosiphum padi* (L.) nymphs and adults (as prey for coccinellids) on barley leaves in arenas with either coccinellid species and with or without *S*. *invicta* workers. The presence of *S*. *invicta* reduced aphid predation by *C*. *maculata* but not *Sc*. *creperus*. The frequency of *S*. *invicta* attack was greater for *C*. *maculata* than *Sc*. *creperus*; mortality was significantly greater for *C*. *maculata* than *Sc*. *creperus*. The wax covering on *Sc*. *creperus* reduced *S*. *invicta* aggression. Unexpectedly, *Sc*. *creperus* larvae with the wax cover removed did not suffer more *S*. *invicta* attacks or increased mortality. In conclusion, the wax cover and, perhaps, the volatile or non-volatile compounds in the wax and on the integument of *Sc*. *creperus* larvae reduce *S*. *invicta* aggression. Further research could identify the wax compounds and determine their functionality as semiochemicals against *S*. *invicta*.

## 1. Introduction

The biological control of hemipterans, such as aphids, mealybugs, and scale insects, by coccinellids has been hampered by tending ants or aggressive, invasive ants foraging for food [1,2]. Ants tend hemipterans (e.g., aphids, and mealybugs) to obtain honeydew [3,4]. Aggressive species, such as the imported fire ant *Solenopsis invicta* Buren (Hymenoptera: Formicidae), attack and kill coccinellid larvae that attempt to prey on tended aphids or mealybugs [5]. *Solenopsis invicta* is known to tend to the mealybug *Phenacoccus solenopsis* Tinsley (Hemiptera: Pseudococcidae) and defend them from predation by the coccinellid *Menochilus sexmaculata* F. [6]. In cotton and soybean fields, *S*. *invicta* workers attack pests and beneficial insects, including predators, e.g., coccinellids [1]. In laboratory experiments, *S*. *invicta* workers attacked and killed 3rd instars and adults of the coccinellid *Hippodamia convergens* Guerin-Meneville on cotton plants infested with cotton aphid *Aphis gossypii* Glover; 3rd instar larvae of *Scymnus louisianae* Chapin were attacked but occasionally escaped predation [5].

Coccinellids may or may not possess effective behavioral, morphological, or chemical defense mechanisms to thwart attacks from aggressive ants [7]. Species in several tribes, i.e., Scymnini, Hyperaspidini, Brachiacanthadini, Ortaliini, Coccidulini, and Azyini, secrete wax from pores in their integument; the wax then forms a covering over their integument [8,9,10,11,12]. The wax covering in *Scymnus nubilus* Mulsant larvae serves to reduce intraguild predation [13]. The wax covering in *Scymnus interruptus* (Goeze) and *Scymnus nigrinus* Kugelann larvae attenuated aggression by the ants *Lasius niger* L. and *Formica polyctena* Forster, respectively [14]. Wax structures on the integument of *Sc. louisianae* reduced aggression by the ant *Lasius neoniger* Emery [15] and chemical cues in the wax could play a role in this modification of ant behavior [15,16]. Species in other tribes, such as Coccinellini, that do not secrete wax could be more susceptible to ant aggression [12]. Note that some coccinellids are myrmecophilous, e.g., *Coccinella magnifica* Redtenbacher, which co-exist with *Formica* spp., in their nests [17,18].

*Scymnus creperus* Mulsant (Coleoptera: Coccinellidae: Scymnini) is native to North America and distributed primarily in the southern regions of the USA [19]. It is a predator of aphids [20,21]. The larvae produce a sticky, white-colored wax that exudes from their integument’s pores. *Coleomegilla maculata* (DeGeer) (Coleoptera: Coccinellidae: Coccinellini) is native to North, Central, and South America [19,22]. It is a predator of aphid nymphs, adults, and lepidopteran and coleopteran eggs [23,24,25]. The larvae do not produce a wax covering but have bristle-like setae that cover their integument. The imported fire ant *S*. *invicta* is native to South America but is well established in the southern USA and northeastern Mexico and parts of the western USA [26,27]. 

Our preliminary research on aphid control by mass-reared *C*. *maculata* larvae has been challenged by the occupation of potted strawberry plants by ants, including *S*. *invicta*. In contrast, the larvae of an unidentified *Scymnus* species attacked and consumed sugarcane aphid *Melanaphis sacchari* (Zehntner) on potted sorghum plants in the presence of tending *S*. *invicta* in Stoneville, Mississippi (EWR, unpublished observations). This study tested the hypothesis that *Sc*. *creperus* larvae are less susceptible than *C*. *maculata* larvae to *S*. *invicta* aggression. The objectives of this study were to determine the predation potential of *C*. *maculata* and *Sc*. *creperus* larvae in the presence or absence of *S*. *invicta* workers and estimate the frequency of *S*. *invicta* attacks and incidence of mortality to *C*. *maculata*, *Sc*. *creperus*, or *Sc*. *creperus* with the wax covering removed. This research will help select the most effective coccinellid species for aphid biological control on crop plants in areas where *S*. *invicta* and other aggressive ants are problematic.

## 2. Materials and Methods

### 2.1. Plant and Insect Cultures

Barley seedlings infested with bird cherry-oat aphid *Rhopalosiphum padi* (L.) nymphs and adults (in banker plant system) were purchased from IPM Laboratories Inc., Locke, NY, USA (https://www.ipmlabs.com). Barley seeds were also purchased from IPM Laboratories to establish barley host plants to maintain a continuous culture of *R*. *padi* at the National Biological Control Laboratory (NBCL), ARS, USDA in Stoneville, MS, USA.

The *Coleomegilla maculata* 3rd instars used in this study were reared from eggs collected randomly from a stock culture maintained in an environmental rearing room (22–24 °C, 45–55% RH, and 16L: 8D L:D photoperiod) in the NBCL. This culture has been maintained for over 30 consecutive generations without any introduction of “feral” individuals. Larvae and adults were reared on factitious food based on a proprietary mixture of brine shrimp (*Artemia franciscana* Kellogg) eggs, microalgae (*Chlorella vulgaris* Beijerink), and fatty acids [28,29].

*Scymnus creperus* adults were purchased from IPM Laboratories Inc. Additional adults were obtained from an established laboratory colony at the University of Florida, in Gainesville, FL, USA. The adults and larvae were fed *R*. *padi* nymphs and adults, which had been reared on barley seedlings in the laboratory, for one generation before experimentation. Just before the experiments, the *R*. *padi* nymphs and adults, 3rd instar *C*. *maculata*, and 3rd instar *Sc*. *creperus* were maintained in a plant growth chamber (22 °C, 60% RH, and 16:8 L:D photoperiod).

Imported fire ants *S*. *invicta* were collected near roadsides and agricultural fields in Washington Co., Stoneville, MS, USA, in the spring and summer of 2018. Fire ants are mound-building ant species. Ants with mound soil were collected by shoveling mound soil into 5-gallon buckets. The water-drip method was then used to separate the ants from the soil [30]. Queen-right colonies were established in rearing trays and maintained in an environmental rearing room (22–24 °C, 45–55% RH, and 16: 8 L:D photoperiod). Distilled water in glass vials, stoppered with cotton, was always provided. Frozen, then thawed house crickets *Acheta domesticus* (L.) were used as a food source. Before experimentation, rearing trays were held on the laboratory benchtop and *S*. *invicta* workers were gently removed and placed in smaller containers coated with fluon. Workers actively moving around in the containers were selected at random for experiments.

### 2.2. Expt. 1: Estimating Predation of R. padi by C. maculata or Sc. creperus in Arenas with or without S. invicta

Using a completely randomized design, this laboratory experiment was set up to estimate predation, i.e., the killing capacity, of 3rd instar *C*. *maculata* or *Sc*. *creperus* on *R*. *padi* nymphs and adults on barley leaves in arenas with or without *S*. *invicta* workers. *Rhopalosiphum padi* mid-to-late instar nymphs and adults were randomly selected from colony cages and placed on a barley leaf in the center of 10 arenas (plastic Petri dishes; 2.5 cm high, 9.0 cm diam., 159 cm^3^, with screened lids). Because 3rd instar *C*. *maculata* are larger than 3rd instar *Sc*. *creperus* (and can purportedly consume more prey), 60 and 50 *R*. *padi* were randomly placed in arenas in the *C*. *maculata* and *Sc*. *creperus* trials, respectively. In preliminary observations, these prey densities exceeded the 7-h killing rate of the coccinellids. Next, five *S*. *invicta* workers, presumably of approximately the same age but variable in size, were randomly selected from an in-house colony (collected in Washington Co., Stoneville, MS, USA on 23 May 2018) and carefully added into 5 of the 10 arenas in the *C*. *maculata* tests. Note that 10 *S*. *invicta* workers were used in the *Sc*. *creperus* tests because preliminary observations indicated that *S*. *invicta* were typically not aggressive to *Sc*. *creperus* larvae. In addition, *S*. *invicta* workers had no prior exposure to *C*. *maculata* or *Sc*. *creperus* larvae at any time in the laboratory. Within 1 h, a single 3rd instar *C*. *maculata* or 3rd instar *Sc*. *creperus* was gently added onto the barley leaf in each arena. At approximately 30-min intervals, within a 7-h time frame, the number of *R*. *padi* killed (including partially or completely consumed) by *C*. *maculata* or *Sc*. *creperus* was determined from each arena. This experiment was replicated four times, i.e., four trials, and the total sample size was 40 observations (or arenas) for *C*. *maculata* and *Sc*. *creperus*. Trials with *C*. *maculata* were conducted on 7, 11, 25 June, and 6 July 2018; those with *Sc*. *creperus* were on 16, 27, 31 July, and 9 August 2018. All arenas were maintained in a plant growth chamber (24 °C, 60% RH, and 16 h photophase), but removed on the day of experimentation. Experiments were performed on a laboratory bench at room temperature (23–24 °C, 40% RH).

### 2.3. Expt. 2: Estimating Frequency of S. invicta Attack and Mortality of C. maculata or Sc. creperus

Using a completely randomized design, this laboratory experiment was carried out to estimate the *S*. *invicta* attack rate and mortality of 3rd instar *C*. *maculata* versus 3rd instar *Sc*. *creperus*. Approximately 20–30 *R*. *padi* nymphs and adults (randomly selected from the colony) were placed on a barley leaf, positioned inside a medium-sized Petri dish arena (2.5 cm high, 9.0 cm diam., 159 cm^3^, with screened lids), and replicated 10 times; five arenas had one *C*. *maculata* and the other five arenas had one *Sc*. *creperus*. Next, 10 *S*. *invicta* workers (randomly selected from an in-house colony established from workers and queens collected in Washington Co., on 23 May 2018) were gently added to each arena. Note that the *S*. *invicta* workers had no prior exposure to *C*. *maculata* or *Sc*. *creperus* larvae at any time in the laboratory. Within 1 h, a single 3rd instar *C*. *maculata* or 3rd instar *Sc*. *creperus* was gently added onto the barley leaf in each arena. At approximately 30-min intervals, the number (and proportion) of *C*. *maculata* versus *Sc*. *creperus* attacked and subsequently killed by *S*. *invicta* was recorded over a 7-h time frame. This experiment was replicated three times, i.e., three trials, and the total sample size was 30 observations (or arenas). Trial dates were on 1, 2, and 3 August 2018. All arenas were maintained in a plant growth chamber (24 °C, 60% RH, and 16 h photophase). On the day of experimentation, the arenas were removed and the experiments were performed on a laboratory bench at room temperature (23–24 °C, 40% RH).

### 2.4. Expt. 3: Frequency of Attack by S. invicta and Mortality of C. maculata, Sc. creperus, or Sc. creperus with Wax Removed

This experiment was identical to Expt. 2, except an additional treatment was added to determine if removing the waxy material from the integument of *Sc*. *creperus* affected the *S*. *invicta* attack rate and mortality of *Sc*. *creperus*. The wax filaments on the dorsal and lateral integument of sample 3rd instar *Sc*. *creperus* were carefully removed with a fine camel’s hair paintbrush. The three coccinellid treatments in this experiment were 3rd instar *C*. *maculata*, 3rd instar *Sc*. *creperus*, or 3rd instar *Sc*. *creperus* without wax. The prey density (20–30 *R*. *padi*) and arenas (medium-sized Petri dishes) were the same as before. The *S*. *invicta* density was 10 workers per arena for all three treatments. The procedures of adding *R*. *padi* onto a barley leaf, then *S*. *invicta* into each arena were the same. At approximately 30-min intervals, the numbers (and proportion) of *C*. *maculata* versus *Sc*. *creperus* versus *Sc*. *creperus* without wax attacked and subsequently killed by *S*. *invicta* were recorded over a 7-h time frame. A total of 5 replicate arenas were used for each treatment, for 15 arenas per trial. Trial dates were 16, 17, and 21 August 2018. The three trials resulted in a sample size of 45 observations (i.e., 45 arenas) for the entire experiment. All arenas were maintained in a plant growth chamber (24 °C, 60% RH, and 16 h photophase). On the day of experimentation, the arenas were removed, and the experiments were conducted on a laboratory bench at room temperature (23–24 °C, 40% RH).

### 2.5. Statistical Analysis

Before analysis, all data were checked for normality (using the Shapiro–Wilk test) and equal variances. For almost all datasets, the assumptions of normality and equal variances were met. Therefore, the square root transformation of absolute data and arcsine transformation of proportional data were not performed. In separate experiments, a Student’s *t*-test was used to test the significance of *S*. *invicta* presence in arenas on predation of *R*. *padi* by *C*. *maculata* or *Sc*. *creperus*. A Pearson Product Moment Correlation Analysis was used to detect any correlation between the approximate time (within a 7-h time frame) that *S*. *invicta* killed *C*. *maculata* and the number of *R*. *padi* killed by *C*. *maculata*. A Student’s *t*-test was used to test the significance of *S*. *invicta* attack frequency and subsequent mortality of *C*. *maculata* and *Sc*. *creperus* in the same experiment. A one-way analysis of variance (one-way ANOVA) was used to test the significance of the *S*. *invicta* attack frequency and subsequent mortality of *C*. *maculata*, *Sc*. *creperus*, or *Sc*. *creperus* without wax covering in the same experiment. The Holm–Sidak Method was used after the one-way ANOVA to separate the mean values. Means were considered significantly different at *p* < 0.05. Computer software programs SigmaPlot 12.0 interfaced with SigmaStat (SYSTAT Software, Inc., San Jose, CA, USA, 2010) and JMP^®^ 14.2.0 (SAS Institute, Inc., Cary, NC, USA, 2018) were used for data analysis.

## 3. Results

### 3.1. Expt. 1: Predation of R. padi by C. maculata or Sc. creperus in the Presence/Absence of S. invicta

**Figure 1:**
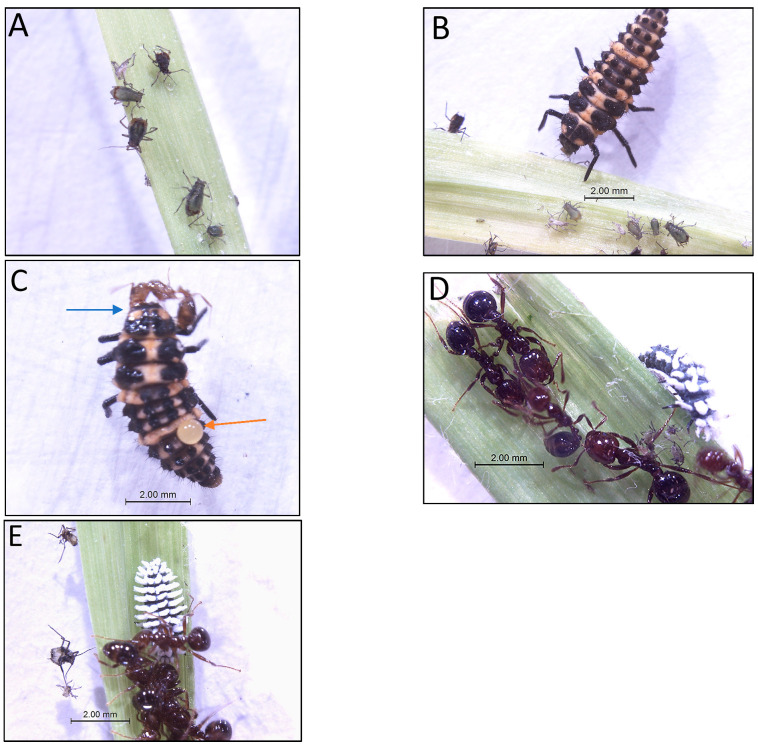
(**A**–**E**) Images of *R*. *padi* nymphs and adults on barley leaf (**A**) and other *R*. *padi* individuals consumed by 3rd instar *C. maculata* (**B**). Image of *S. invicta* worker attacking 3rd instar *C. maculata* (**C**) in the arena. The blue arrow illustrates *S. invicta* biting *C. maculata*; the orange arrow illustrates a drop of hemolymph exuding from the integument of *C. maculata* after being stung by *S. invicta*. Images of 3rd instar *Sc. creperus* not being attacked by *S. invicta* workers (**D**,**E**).

Figure 1 illustrates *R*. *padi* nymphs and adults on a barley leaf. Companion images illustrate *R*. *padi* being consumed by 3rd instar *C. maculata*, *S. invicta* stinging and biting 3rd instar *C. maculata*, and *S. invicta* not attacking 3rd instar *Sc. creperus*.

**Figure 2:**
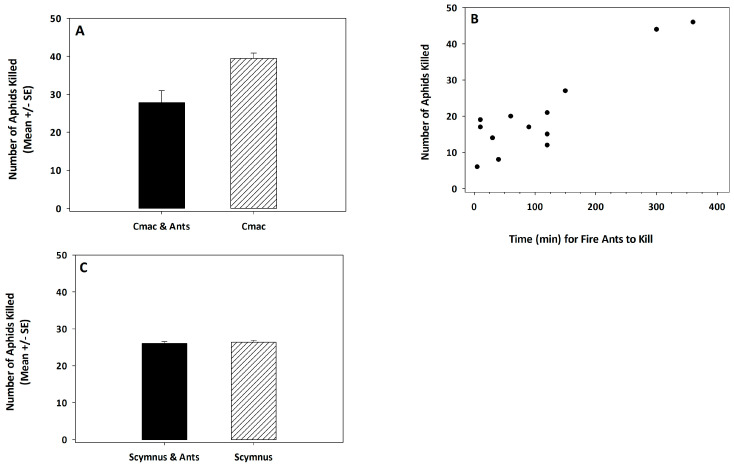
The mean ± SE number of aphids killed by 3rd instar *C. maculata* in the presence or absence of *S. invicta* (**A**), and a scatterplot of the number of aphids killed by *C. maculata* versus the time (min) required for *S. invicta* to kill *C. maculata* (**B**). The mean ± SE number of aphids killed by 3rd instar *Sc. creperus* in the presence or absence of *S. invicta* (**C**). The sample size was 40 observations for the test with *C. maculata* and the test with *Sc. creperus*. The scatterplot represented 13 observations.

The mean ± SE number of *R. padi* killed by 3rd instar *C. maculata* was significantly lower in the presence of *S. invicta* (*t* = 3.32, df = 38, *p* = 0.002; Figure 2A). *Solenopsis invicta* did not kill or tend *R. padi* nymphs or adults. The mean (± SE) proportion of *R. padi* killed during the 7-h test period was 0.46 (± 0.05) and 0.66 (± 0.02) in the *C. maculata* and *S. invicta* treatment versus *C. maculata* alone treatment, respectively. There were 60 *R. padi* nymphs and adults in each arena at the onset of the experiment with *C. maculata*. *Solenopsis invicta* worker aggression reduced the predation capacity of *C. maculata*. Moreover, the number of *R. padi* killed by *C. maculata* decreased as the time required for *S. invicta* to detect and kill *C. maculata* decreased (*R*_c_ = 0.898; *p* < 0.001; n = 13 observations; Figure 2B).

The mean ± SE number of *R. padi* killed by 3rd instar *Sc. creperus* was not affected by the presence of *S. invicta* (*t* = 0.45, df = 38, *p* = 0.651; Figure 2C). Moreover, the mean (±SE) proportion of *R. padi* killed during the 7-h test period was 0.52 (±0.009) and 0.53 (±0.01) in the *Sc. creperus* and *S. invicta* treatment or *Sc. creperus* alone, respectively. There were 50 *R. padi* nymphs and adults in each arena at the onset of the experiment with *Sc. creperus*. Generally, *S. invicta* workers were not aggressive to *Sc. creperus*; no *Sc. creperus* were killed in any of the arenas.

### 3.2. Expt. 2: Frequency of Attack by S. invicta and Mortality of C. maculata or Sc. creperus

**Figure 3:**
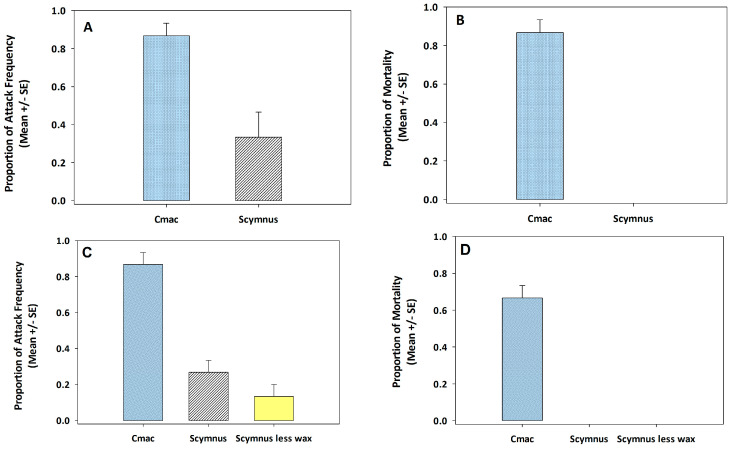
The mean ± SE proportional frequency of attack by *S. invicta* and mortality of *C. maculata* or *Sc. creperus* (**A**,**B**). The sample size was six observations for proportional attack frequency and proportional mortality estimates for *C. maculata* or *Sc. creperus* with or without *S. invicta*. The mean ± SE proportional frequency of attack by *S. invicta* and mortality of *C. maculata*, *Sc. creperus*, or non-wax covered *Sc. creperus* (**C**,**D**). The sample size was nine observations for proportional attack frequency, and mortality estimates for *C. maculata*, *Sc. creperus*, or non-wax-covered *Sc. creperus* with or without *S. invicta*.

As illustrated in Figure 3A,B, *S. invicta* attacked and killed 3rd instar *C. maculata* rather than *Sc. creperus* in experimental arenas. The attack frequency was significantly greater on *C. maculata* than *Sc. creperus* (*t* = 3.58, df = 4; *p* = 0.023). Mortality resulting from *S*. *invicta* attacks was significantly different between *C. maculata* and *Sc. creperus* (*t* = 13, df = 4; *p* < 0.001). The attack frequency and mortality of *C. maculata* were approximately the same, indicating that *S. invicta* killed approximately all the *C. maculata* they attacked. The mortality of 3rd instar *C. maculata* was from the biting and stinging behavior of *S. invicta*. Moreover, in any of the three trials, *S. invicta* never consumed *C. maculata* during the 7-h test period. Although *S. invicta* attacked a small proportion of *Sc. creperus* during the 7-h test period (Figure 3A), none of them were killed (Figure 3B). *Solenopsis invicta* workers were seen biting the wax filaments covering the integument of 3rd instar *Sc. creperus*. Several seconds later, *S. invicta* released its grip on *Sc. creperus* and immediately began to rub its mandibles against the substrate, attempting to remove the wax particles.

### 3.3. Expt. 3: Frequency of Attack by S. invicta and Mortality of C. maculata, Sc. creperus, or Sc. creperus with Wax Removed

The artificial removal of the wax covering from 3rd instar *Sc. creperus* before exposure to *S. invicta* workers did not increase the attack frequency. It was greatest on *C. maculata* than on wax-covered or non-wax-covered *Sc. creperus* (*F* = 13.95, df = 2, 6; *p* = 0.006; Figure 3C). Moreover, significant differences were not detected in attack frequency between wax-covered and non-wax-covered *Sc. creperus*. Removal of the wax filaments from the integument of *Sc. creperus* did not increase the mortality of *Sc. creperus*. Attacks from *S. invicta* resulted in the mortality of *C. maculata* but never of *Sc. creperus* (*F* = 169.63, df = 2, 6; *p* < 0.001; Figure 3D).

## 4. Discussion

This laboratory study clearly observed the differential susceptibility of *C*. *maculata* versus *Sc*. *creperus* to *S*. *invicta* aggression. The predation capacity of *C*. *maculata* larvae was reduced by *S*. *invicta* attacks and subsequent mortality. In contrast, the predation capacity of *Sc*. *creperus* was not affected by *S*. *invicta*. In previous studies, the wax covering the integument of *Scymnus* larvae reduced the aggressive behavior of ants tending the aphids for their honeydew or foraging for food [15,17]. Coccinellids that lack the wax covering, as in the case of *C*. *maculata* and relatives in the tribe Coccinellini, are susceptible to ant aggression. For example, the ant *Monomorium minimum* (Buckley) tended to the soybean aphid *Aphis glycines* Matsumura in the laboratory and consequently hindered predation by the coccinellid *Harmonia axyridis* (Pallas) [2]. Larvae of *Ha*. *axyridis* are devoid of a wax covering and are members of the tribe Coccinellini. In another study, *S*. *invicta* reduced the predation efficiency of *H. convergens* (tribe Coccinellini) 3rd instars more than *Sc. louisianae* (tribe Scymnini) 3rd instars in laboratory experiments with the cotton aphid *A. gossypii* [5]. The susceptibility to ant aggression suggests that biological control success will not be as effective with coccinellid larvae that do not possess a wax cover.

Although *C*. *maculata* larvae possess morphological defenses (spines, or setae) on the dorsal surface of their integument and chemical defenses (alkaloids) in their hemolymph, these defenses provide little protection from *S*. *invicta* aggression. Alkaloids discovered in *C*. *maculata* adults might function as semiochemicals [31,32]. Precoccinellin was thought to defend against some predators [31], whereas 2,4,6-trimethylpyridine may function as a pheromone [32]. To the best of our knowledge, the presence or concentration of either compound in *C*. *maculata* larvae has not been determined.

Wax structures on the integument of *Sc*. *louisianae* larvae provided a morphological and perhaps chemical defense against attacks from the ant *L*. *neoniger*; removal of wax from the integument of *Sc*. *louisianae* larvae did not attenuate *L*. *neoniger* aggression [15]. Dissolving the wax structures in hexane, then applying the solution to the integument of *Sc*. *louisianae* larvae reduced ant aggression, suggesting that chemical cues in the wax were partly responsible for lowering *L*. *neoniger* aggression [15]. In this study, removing the wax covering did not increase *S*. *invicta* attacks or cause mortality of *Sc*. *creperus*, suggesting that chemical compounds on the surface of their integument, in addition to the wax, might play a defensive role, perhaps as a repellent or an irritant. Moreover, *S*. *invicta* workers rubbed their mandibles against the substrate immediately after biting, then released wax-covered *Sc*. *creperus* larvae, apparently cleaning their mandibles. This *S*. *invicta* cleaning behavior further suggests a repellent or irritant functionality of the wax. Future research could identify the chemical compounds in the wax covering and on the surface of the integument of *Sc*. *creperus* larvae. In addition, determining the function of the isolated compounds in bioassays against *S*. *invicta* workers would be a worthwhile investigation potentially leading to the discovery of novel repellents.

Complimentary research to develop effective baits and bio-based insecticides against *S*. *invicta* is needed. Perhaps, combining tactics to control aphids by releasing the most effective coccinellids and using baits and repellents to keep *S*. *invicta* workers away from crop plants in cultured systems, e.g., greenhouses, would be the best approach.

In conclusion, this study provides evidence for the differential susceptibility of *C*. *maculata* and *Sc*. *creperus* larvae to aggressive behavior from *S*. *invicta* workers in laboratory arenas. Wax covering the dorsal and lateral surfaces of the *Sc*. *creperus* integument provides protection and prevents mortality from *S*. *invicta* attacks. The selection of the most effective aphidophagous coccinellids would ensure the success of the biological control of aphids on crop plants. The selection of wax-covered larvae (Scymnini) rather than non-wax-covered larvae (Coccinellini) would be the best choice in areas, such as greenhouses or glasshouses, where aphid-tending ants or aggressive ants foraging for food on or near crop plants are problematic [33].

## Data Availability

At the authors’ discretion, data supporting the article’s results can be made available on ResearchGate.
